# Risk-adapted moderate hypofractionation of prostate cancer: A prospective analysis of acute toxicity, QOL and outcome in 221 patients

**DOI:** 10.1007/s00066-019-01477-y

**Published:** 2019-05-28

**Authors:** Andreas Schörghofer, Michael Groher, Josef Karner, Andrea Kopp, Gerhard Kametriser, Thomas Kunit, Josef Holzinger, Felix Sedlmayer, Frank Wolf

**Affiliations:** 1 Department of Radiotherapy and Radio-Oncology, LKH Salzburg, University Clinics, Paracelsus Medical University, Müllner Hauptstraße 48, 5020 Salzburg, Austria; 2 Department. of Urology, LKH Salzburg, University Clinics, Paracelsus Medical University, Müllner Hauptstraße 48, 5020 Salzburg, Austria; 3 Department of Surgery, LKH Salzburg, University Clinics, Paracelsus Medical University, Müllner Hauptstraße 48, 5020 Salzburg, Austria

**Keywords:** Dose escalation, Hypofractionation, Risk stratification, Spacer, Pelvic lymph nodes, Dosiseskalation, Hypofraktionierung, Risikobeurteilung, Spacer, Pelvine Lymphknoten

## Abstract

**Purpose:**

Prostate cancer (PCA) is highly heterogeneous in terms of its oncologic outcome. We therefore aimed to tailor radiation treatment to the risk status by using three different hypofractionated radiation regimen differing in applied dose, use of rectum spacer, inclusion of pelvic lymph nodes (pLN) and use of androgen deprivation therapy (ADT). Here we report on acute toxicity, quality of life (QOL) and oncologic outcome at a median follow-up of 12 months.

**Methods:**

A total of 221 consecutive PCA patients received hypofractionated intensity-modulated radiotherapy (IMRT). Low-risk (LR) patients were planned to receive 60 Gy in 20 fractions (EQD2^α/β1.5^ = 77.1 Gy), intermediate-risk (IR) patients 63 Gy in 21 fractions (EQD2^α/β1.5^ = 81 Gy), and high-risk (HR) patients 67.5 Gy in 25 fractions (EQD2^α/β1.5^ = 81 Gy) to the prostate and 50 Gy in 25 fractions to the pLN. Acute rectal toxicity was assessed by endoscopy. In addition, toxicity was scored using CTC-AE 4.0 and IPSS score, while QOL was assessed using QLQ-PR25 questionnaires.

**Results:**

Acute CTC reactions were slightly higher in the HR regimen but reverted to baseline at 3 months. GI G2 toxicity was 4%, 0% and 12% for the LR, IR and HR regimen. Compared to IR patients, the increase in toxicity in HR patients was statistically significant (*p* = 0.002) and mainly caused by a higher incidence of diarrhea presumably due to pelvic EBRT. QOL scores of all domains were worse for the HR regimen (not significant).

**Conclusion:**

Risk-adapted moderate hypofractionation is associated with low GI/GU toxicity. Given the higher rate of pelvic metastases in HR patients, slightly higher transient acute reactions should be outweighed by possible oncological benefits.

## Zusammenfassung

**Ziel:**

Das Prostatakarzinom (PCA) ist in Bezug auf das onkologische Ergebnis eine sehr heterogene Erkrankung. Wir etablierten drei risikoadaptierte, hypofraktionierte Bestrahlungsschemata, die sich in der applizierten Dosis, Verwendung von Rektum-Spacern, Einschluss der pelvinen Lymphknoten (pLN) sowie dem Einsatz einer Androgendeprivationstherapie (ADT) unterschieden. Hier berichten wir über die akute Toxizität, Lebensqualität (QOL) sowie das onkologische Ergebnis mit einem medianen Follow-up von 12 Monaten.

**Methoden:**

Eine hypofraktonierte intensitätsmodulierte Radiotherapie (IMRT) erhielten 221 konsekutive PCA-Patienten . Niedrigrisiko(LR)-Patienten erhielten 60 Gy mit 20 Fraktionen (EQD2^α^^/^^β^^1,5^ = 77,1 Gy), Intermediärrisiko(IR)-Patienten 63 Gy mit 21 Fraktionen (EQD2^α^^/^^β^^1,5^ = 81 Gy) und Hochrisiko(HR)-Patienten wurden mit 67,5 Gy mit 25 Fraktionen (EQD2^α^^/^^β^^1,5^ = 81 Gy) auf die Prostata und 50 Gy mit 25 Fraktionen auf die pLN bestrahlt. Die akute rektale Toxizität wurde mittels Endoskopie beurteilt. Weiters wurden CTC-AE V4.0 sowie IPSS-Scores erhoben. Die QOL wurde mittels QLQ-PR25-Fragebögen ermittelt.

**Ergebnisse:**

Die akute Toxizität war in der HR-Gruppe etwas höher, ging allerdings nach 3 Monaten auf das Ursprungsniveau zurück. Die gastrointestinale (GI) G2-Toxizität betrug 4%, 0% und 12% für die LR-, IR- bzw. HR-Gruppe. Verglichen mit den IR-Patienten war der Toxizitätsanstieg der HR-Patienten statistisch signifikant (*p* = 0,002) und wurde hauptsächlich durch die höhere Diarrhoe-Rate verursacht, vermutlich bedingt durch die pelvine EBRT. Die QOL-Werte aller Domänen waren in der HR-Gruppe etwas schlechter (nicht signifikant).

**Schlussfolgerung:**

Risikoadaptiere moderate Hypofraktionierung ist mit einer niedrigen GI/GU-Toxizität assoziiert. Aufgrund der höheren Rate pelviner Metastasen in HR-Patienten sehen wir die höheren transienten Akutreaktionen durch den möglichen onkologischen Benefit gerechtfertigt.

## Introduction

The prognosis of prostate cancer is highly dependent on its risk profile as developed by D’Amico et al. [1] and adopted by the RTOG and AUA. LR patients have a life expectancy similar to healthy individuals, no matter if they are actively treated or actively surveilled [2]. In contrast, HR patients are more prone to develop systemic and/or locoregional progression and are more likely to die from prostate cancer [3, 4].

However, these significant differences in outcome are not necessarily reflected in dose recommendations, although the dose–response relationship is clearly intact even above 80 Gy as shown by numerous dose escalation trials [5] and epidemiologic surveys [6]. NCCN guidelines recommend normofractionated as well as moderately hypofractionated schedules ranging from EQD2 72 to 84.2 Gy irrespective of risk status. German S3 guidelines recommend doses from 74–80 Gy in the setting of normofractionation for all risk groups; for hypofractionation, no explicit dose recommendations are given. This may contribute to a situation where patients of all risk groups receive rather the same radiation schedule in a ‘one size fits all’ manner.

We therefore aimed to tailor radiation treatment to the risk status by using three different radiation regimen which differ in applied dose, fractionation, use of rectum spacers, inclusion of pLN and prescription of ADT.

Hypofractionated treatment regimen were prescribed with doses of EQD2 78 Gy for LR, and 81 Gy for IR and HR patients. In the latter, pLN were treated with 50 Gy with inclusion of the common iliac nodes.

In order to mitigate the expected higher rectal toxicity in IR and HR patients, rectal spacers were implanted via perineal injection into the retroprostatic space behind the Denovillier fascia. Rectal spacers are effective in reducing dose to the anterior rectal wall [7] and have been shown to reduce gastrointestinal toxicity in randomized prospective trials [8, 9].

Here we report on acute toxicity, quality of life and biochemical control after tailored hypofractionated treatment of low, intermediate and high risk prostate cancer patients.

## Materials and methods

### Inclusion criteria and workup

To be eligible for hypofractionated treatment patients had to have an IPSS score <12 and had to be fit for short general anesthesia for the rectal spacer application (except low-risk patients).

Pretherapeutic F‑choline or PSMA-PET-CT was required for HR patients.

### Treatment

Patients were risk stratified in low, intermediate and high risk according to the D’Amico classification.

LR patients were first scheduled for hypofractionated radiotherapy with 63 Gy in 3 Gy fractions (EQD2^α/β1.5^ = 81 Gy) with a spacer (until 11/2016). Thereafter, they received 60 Gy in 3 Gy fractions (EQD2^α/β1.5^ = 77.1 Gy) without a spacer.

IR patients were treated with 63 Gy in 21 fractions (EQD2^α/β1.5^ = 81 Gy). Short-term neoadjuvant/adjuvant androgen deprivation therapy (ADT) was administered for 6 months starting 3 months prior to start of radiotherapy.

HR patients were planned to receive 25 fractions of normofractionated treatment to the pLN (50 Gy/2 Gy single dose), together with a hypofractionated simultaneous integrated boost to the prostate (67.5 Gy/2.7 Gy, EQD2^α/β1.5^ = 81 Gy) and the seminal vesicles (60 Gy/2.4 Gy, EQD2^α/β1.5^ = 66.9 Gy), respectively. If pLN were positive in pretherapeutic PSMA-PET-CT, they were simultaneously boosted with 25 × 2.4 Gy. Long-term ADT was administered for 24–36 months, starting 3 months prior to radiotherapy. Table 1 provides an overview of treatment regimen.

**Table 1:** Treatment regimen overview

	Fractionation	EQD2^α/β1.5^	Spacer	*p* LN	AD	Technique
*Low risk*	20 × 3/21 × 3^a^	77.1 Gy/81 Gy	No/yes^a^	No	No	7-field IMRT
*Intermediate risk*	21 × 3	81 Gy	Yes	No	6 months	7-field IMRT
*High risk*	25 × 2.7/2.4/2 P/SV/pLN	81/66.9/50 Gy P/SV/pLN	Yes	Yes	24 months	VMAT dual arc

^a^31 patients treated before November 2016

In all patients, gold fiducials were placed for image guidance (IGRT). In addition, IR and HR patients received either a gel (*SpaceOAR*^TM^, Augmenix Inc., Waltham, MA, USA) or a balloon spacer (*ProSpace*^TM^, BioProtect Inc., Kfar-Saba, Israel) prior to the planning CT.

Planning CT and MRI were performed the same day and fused based on the implanted gold fiducials. For the planning MRI, a turbo field echo sequence was used, optimized to visualize metal artefacts, anatomical (prostate) and liquid (spacer) structures. Prior to image acquisition, patients were instructed to have a full bladder and empty their bowels. Routine use of mild laxatives was recommended.

Contouring of the prostate CTV was performed on the MR in transversal plane, aided by sagittal and coronary plane contours when needed.

PET-positive lymph nodes were identified on the fused PET-CT and contoured as separate volumes on the planning CT.

PTV margins of the prostate were 6 mm in all directions. PTV margins of pLN target volumes were adapted to accommodate prostate movements as published previously [10]. In short, margins were 10 mm, 10 mm and 5 mm in sup/inf, ant/post and lateral directions in order to compensate for mismatch to the bony anatomy resulting from referencing pelvic fields to gold fiducials in the prostate.

For EBRT, LR and IR patients were treated using 7‑field IMRT, HR patients using dual arc VMAT technique, respectively.

For EBRT, a minimum bladder volume was defined based on the filling status of the planning CT and verified prior to each fraction using an ultrasound bladderscan device (*Uscan*, Signostics, London, UK). Daily IGRT was routinely carried out by registering gold fiducials using two orthogonal kilovolt images, typically at 0 and 90° as described previously [11].

### Toxicity assessment

Rectal toxicity was assessed by performing endoscopy and scoring the rectal mucosa based on 5 domains using the VRS ([12]; Table 2).

**Table 2:** Vienna Rectoscopy Score (VRS)

VRS	Congested mucosa	Telangiectasia	Ulceration	Stricture	Necrosis
Score 0	Grade 1	None	None	None	None
Score 1	Grade 2	Grade 1	None	None	None
Score 2	Grade 3	Grade 2	None	None	None
Score 3	Any	Grade 3	Grade 1	None	None
Score 4	Any	Any	Grade 2	Grade 1	None
Score 5	Any	Any	Grade ≥3	Grade ≥2	Any

Endoscopy and CTC scoring were performed at day 1 after implantation, at the end of RT, and 12 months thereafter or at any time the patient reported rectal complaints.

In addition, toxicity was assessed using CTCAE v4.0 scoring for the following gastrointestinal and urogenital domains: hematuria, urinary frequency, incontinence, retention and urgency, diarrhea, fecal incontinence, proctitis, rectal hemorrhage and rectal ulcer. For analysis, the highest grade of any gastrointestinal (GI) and any urogenital (GU) domain was scored.

Quality of life was assessed using QLQ PR-25 questionnaires. PSA measurements, CTC scoring, IPSS scoring, and clinical exam were performed at start of RT, end of RT, and at 3, 6 and 12 months post RT, respectively. Subscores of QLQ-PR25 were calculated as recommended in the scoring manual and reported for urinary symptoms, bowel symptoms, hormone treatment-related symptoms and sexual function scores.

### Statistical analysis

The Student’s t‑test was used to analyze differences in QLQ and IPSS scores between treatment groups. To determine whether the data are normally distributed, the Kolmogorov–Smirnov test was applied. A Mann–Whitney U test was conducted if the data were not normally distributed and for the CTC scores. Spearman’s rho was computed to assess the correlation between the IPSS and urinary toxicity scores.

## Results

### Patient characteristics

From February 2015 to July 2018, 221 patients referred for primary radiotherapy were eligible to receive risk adapted hypofractionated radiation treatment (Table 3 for patient characteristics).

**Table 3:** Patient characteristics

	*n*	%
Number of patients	221	–
Age (mean)	75	–
Neoadjuvant hormonal therapy	149	67
T stage		
T1a	2	1
T1b	1	0
T1c	120	54
T2	15	7
T2a	19	9
T2b	10	5
T2c	16	7
T3	6	3
T3a	1	0
T3b	1	0
T4	1	0
PSA concentration (ng/mL)		
<10	130	59
10–20	67	30
>20	24	11
Gleason score		
≤6	108	49
7	64	29
≥8	38	17
Risk group		
Low	64	29
Intermediate	96	43
High	61	28

*PSA* prostate-specific antigen

Risk groups according to D’Amico classification were well balanced with 31%, 42% and 27% for LR, IR and HR, respectively.

In all, 100% of LR, 95% of IR and 83% HR patients were treated per protocol. The first 31 LR patients (48%) were treated with 21 × 3 Gy with a spacer, similar to IR patients. Since all statistical analyses aim at morbidity, toxicity was analyzed according to the respective treatment escalation group so that the categorization “LR”, “IR” and “HR” refers to the given treatment and not to the initial D’Amico risk classification.

In 8 HR patients (13.1% of HR patients), PET-positive lymph nodes were identified and boosted with 60 Gy in 2.4 Gy fractions.

### Toxicity

**Fig. 1:**
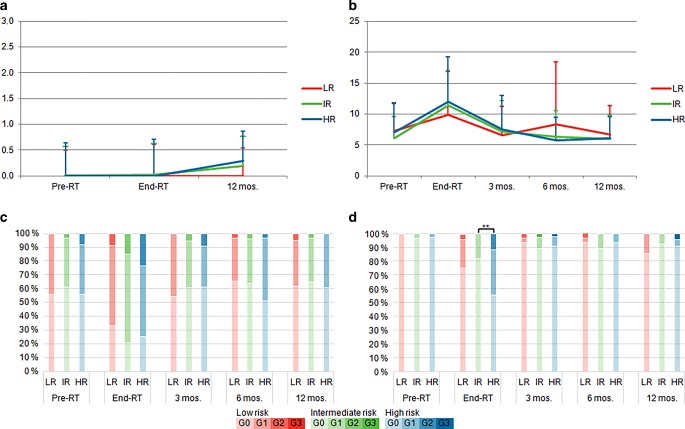
Acute toxicity. Shown are Vienna Rectoscopy Scores (**a**), IPSS scores (**b**) and acute CTC toxicity for the urogenital (**c**) and gastrointestinal (**d**) domain for the low risk (LR), intermediate risk (IR) and high risk (HR) regimen, respectively. * = *p* ≤ 0.05, ** = *p* ≤ 0.01, *** = *p* ≤ 0.001

Vienna rectoscopy scores (VRS, Fig. 1a): Acute rectal toxicity was assessed by performing endoscopy and scoring mucosal alterations.

Rectal scores were very low in all risk groups. Average scores at end of RT and after 12 months were 0.01 and 0.2, respectively. There was no significant difference between LR, IR and HR regimen patients at any time point.

IPSS scores (Fig. 1b): Average IPSS score at baseline was 6.5. At the end of radiotherapy, IPSS deteriorated somewhat to 10.0, 11.6 and 12.3 for LR, IR and HR regimen patients, respectively, but recovered to baseline levels after 3 months. There was no significant difference between the regimen.

CTCAE v. 4.0 scores (Fig. 1c, d): Acute GU G1 toxicity was 58%, 64% and 51% for LR, IR and HR regimen at the end of RT and returned to baseline levels at 3 months. Acute GU G2 toxicity was very low overall with 10% at the end of RT (8%, 15% and 23% for LR, IR and HR) and returned to baseline at 3 months. No G3 toxicities were reported.

Acute GI G1 toxicity at end of RT was 21%, 18% and 33% for LR, IR and HR, respectively, and reverted to near baseline levels at 3 months. GI G2 toxicity was 4%, 0% and 12%. The increase in toxicity in high-risk patients was statistically significant (*p* = 0.002) compared to IR and mainly caused by a higher incidence of diarrhea. One HR patient experienced late G3 toxicity at 12 months (rectal hemorrhage). Three patients experienced rectal spacer perforation at 2–3 months after radiotherapy resulting from a faulty spacer placement into the rectal wall. After endoscopic removal of the spacer balloon, the perforation healed without any further therapy or complications. For more information, spacer-related toxicities have recently been published by our group [13].

### Quality of life

**Fig. 2:**
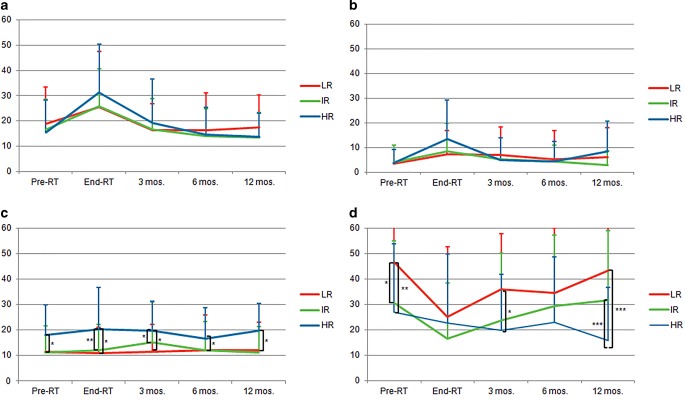
Quality of life scores for the urinary (**a**), bowel (**b**), hormonal treatment-related symptoms (**c**) and sexual functioning (**d**) domains. *RT* radiotherapy, *mos* months; *LR* low risk, *IR* intermediate risk, *HR* high risk. * = *p* ≤ 0.05, ** = *p* ≤ 0.01, *** = *p* ≤ 0.001

Quality of life (QOL) scores are illustrated in Fig. 2. Urinary subscore of PR25 questionnaire for all risk groups had an average baseline level of 16.7. By the end of therapy, the urinary score increased to a comparable extent in all regimen groups (27.1) but reverted to baseline at 6 months after the end of therapy. This is in good concordance with the acute toxicity assessment which showed very similar dynamics. There was a strong correlation of pretherapeutic IPSS score and maximum urinary toxicity score (r = 0.430, *p* ≤ 0.01).

Bowel score was low at baseline (3.8) and increased in all risk groups at the end of treatment (9.4). In HR patients the increase was slightly more pronounced, but the difference failed to reach significance compared to LR (*p* = 0.068) and IR (*p* = 0.090).

Hormonal treatment-related symptoms were significantly higher in HR patients at all time points. Sexual functioning was significantly worse in HR patients.

### Outcome

**Fig. 3:**
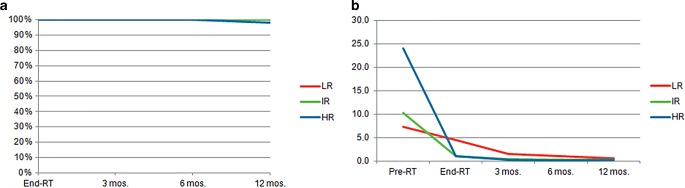
Biochemical control. Kaplan–Meier analysis of biochemichal progression-free survival (**a**) and average PSA values (**b**) for LR, IR and HR patients

The Kaplan–Meier analysis is shown in Fig. 3. Average pretreatment PSA was 7.3 ng/ml, 10.2 ng/ml and 24.1 ng/ml for LR, IR and HR patients, respectively. PSA of IR and HR patients rapidly declined until the end of RT (1.1 and 1.0 ng/ml) as a result of ongoing ADT. PSA values of LR patients continued to decrease to 0.6 ng/ml at 12 months. Biochemical progression-free survival at a median follow-up of 12 months was 99% overall, and 100%, 100% and 97% for LR, IR and HR patients, respectively.

## Discussion

We have compared toxicity profiles of three different hypofractionation schedules which were designed under the assumption that HR prostate cancer requires escalated treatment compared to LR prostate cancer which holds a high risk of overtreatment. Treatment differed in applied dose, use of rectal spacers, use of antihormonal medication and inclusion of pLN.

We could show that all treatment regimen were in total tolerated well in terms of acute toxicity, scored by both, CTC and direct rectoscopic inspection of the rectal mucosa. However, the HR regimen featured slightly higher toxicity in nearly all assessed domains as well as quality of life. The difference was statistically significant in GI CTC compared to IR regimen. This higher GU and GI toxicity can mainly be attributed to the inclusion of pLN, which inevitably causes higher doses to the small bowel, rectum and bladder. Of note, in our series all toxicity domains reverted to near baseline levels after 6 months. To our mind, this slightly higher acute toxicity is outbalanced by the benefit of treating possible occult nodal disease in HR patients. Since the absolute toxicity was very low and only transient, no severe late toxicities are to be expected as consequential late effects [14], but this will be subject of longer follow-up. For scoring of mucosal reactions, the VRS was used which was designed to classify acute and late mucosal reactions and was validated with the EORTC classification of late radiation reactions. Using endoscopic scoring of rectal toxicities allowed the detection of clinically inapparent mucosal reactions.

The benefit of pLN irradiation is heavily debated [15] since the scarce and somewhat outdated randomized data do not provide clear evidence for an improvement in either overall or cancer-specific survival [16–18]. However, these trials have in part been criticized for using inappropriate doses and insufficient field sizes. In addition, Spratt et al. recently showed that the common iliac lymph nodes are a site of frequent locoregional failure, and that these subvolumes are not typically covered when adhering to RTOG contouring guidelines [19]. These shortcomings might in part explain why no clear benefit for pLN irradiation has yet been demonstrated in prospective trials. However, experiences from other tumor entities suggest that pLN irradiation reduces regional failures and might improve overall survival.

The advent of PSMA PET CT provided a superior specificity and sensitivity in detection of lymph node metastases [20], thus, building a possible rationale to spare pelvic irradiation in patients who are node negative in PSMA PET-CT. This concept has to be evaluated in prospective trials.

For LR and IR prostate cancer, moderate hypofractionation has been shown to be non-inferior to normofractionated treatment in several prospective randomized trials [21–24] and is now recommended in the primary setting by NCCN guidelines [25], amongst others. For HR patients, the benefit of hypofractionated radiotherapy is less clear. However, results from three large meta-analyses suggests that the low α/β ratio is an intrinsic property of all prostate cancer cells irrespective of their Gleason score or grading [26]. The same is true for dose escalation in the setting of long-term androgen deprivation. While there is no clear consensus that the dose–effect relationship is intact at doses above 80 Gy in the presence of ADT, several analyses point well in that direction [27, 28]. We have therefore opted to treat patients of all risk groups using moderate hypofractionation schedules for the prostate and normofractionation to the pLN in HR patients.

Although the applied dose to the prostate in IR and HR patients (EQD2 of 81 Gy assuming an α/β ratio of 1.5 Gy) was slightly higher than in the above mentioned hypofractionation trials, the observed toxicity compares favorably with a very low incidence of G2 GI and GU toxicity. We could not detect any differences in toxicity between the 20 × 3 Gy and the 21 × 3 Gy arm. The low rectal toxicity of the latter might in part be attributed to the rectal spacer.

In terms of oncologic outcome, follow-up is too short to draw any conclusions, but the bPFS of 99% at 12 months lies within the expected range.

To our knowledge, this is the first comparison of risk adapted tailored hypofractionated treatment regimen with or without inclusion of the pLN which provides insight on added combined toxicity in the context of a modern and risk-adapted hypofractionated regimen.

In HR patients, slightly higher transient acute reactions should be outweighed by possible oncological benefits.
